# Closing the gap between screening and depression prevention: a qualitative study on barriers and facilitators from the perspective of public health professionals in a school-based prevention approach

**DOI:** 10.1186/s12889-023-15705-9

**Published:** 2023-05-12

**Authors:** Marloes W. G. Braam, Sanne P. A. Rasing, Dewi A. M. Heijs, Joran Lokkerbol, Diana D. van Bergen, Daan H. M. Creemers, Jan Spijker

**Affiliations:** 1 Behavioural Science Institute, Radboud University, Nijmegen, the Netherlands; 2 GGZ Oost Brabant, Oss, the Netherlands; 3 Trimbos Institute, Utrecht, the Netherlands; 4 University of Groningen, Groningen, the Netherlands; 5 Pro Persona, Nijmegen, the Netherlands

**Keywords:** Depressive symptoms, Adolescents, Screening, Prevention, School-based, Public health professional

## Abstract

**Background:**

The prevalence of depression has increased among adolescents in western countries. Prevention is needed to reduce the number of adolescents who experience depression and to avoid negative consequences, including suicide. Several preventive interventions are found to be promising, especially multi-modal approaches, for example combining screening and preventive intervention. However, an important bottleneck arises during the implementation of preventive intervention. Only a small percentage of adolescents who are eligible for participation actually participate in the intervention. To ensure that more adolescents can benefit from prevention, we need to close the gap between detection and preventive intervention. We investigated the barriers and facilitators from the perspective of public health professionals in screening for depressive and suicidal symptoms and depression prevention referral in a school-based setting.

**Methods:**

We conducted 13 semi-structured interviews with public health professionals, who execute screening and depression prevention referral within the Strong Teens and Resilient Minds (STORM) approach. The interviews were recorded, transcribed verbatim, and coded in several cycles using ATLAS.ti Web.

**Results:**

Three main themes of barriers and facilitators emerged from the interviews, namely “professional capabilities,” “organization and collaboration,” and “beliefs about depressive and suicidal symptoms and participation in prevention”. The interviews revealed that professionals do not always feel sufficiently equipped in terms of knowledge, skills and supporting networks. Consequently, they do not always feel well able to execute the process of screening and prevention referral. In addition, a lack of knowledge and support in schools and other cooperating organizationorganizations was seen to hinder the process. Last, the beliefs of public health professionals, school staff, adolescents, and parents —especially stigma and taboo—were found to make the screening and prevention referral process more challenging.

**Conclusions:**

To further improve the process of screening and prevention referral in a school-based setting, enhancing professional competence and a holding work environment for professionals, a strong collaboration and a joint approach with schools and other cooperating organizations and society wide education about depressive and suicidal symptoms and preventive intervention are suggested. Future research should determine whether these recommendations actually lead to closing the gap between detection and prevention.

## Background

Depression is one of the most common mental disorders among adolescents. In recent years the prevalence of depression has increased among adolescents in western countries [1]. Depression and even subclinical depressive symptoms [2] have many adverse impacts on adolescents and the society, including suicide attempts and suicide, recurrent episodes later in life, and very high economic and social consequences [3–9]. Prevention is needed to reduce the number of adolescents who experience this mental disorder, which forms a substantial part of the world’s burden of disease [7].

Preventive mental health interventions exist at three different levels: universal prevention, which is aimed at all individuals; selective prevention, which focuses on individuals at risk; and indicated prevention, which targets individuals with elevated symptoms. Despite some limitations, several preventive interventions have been found to be effective in reducing depressive symptoms and reducing the risk of depression diagnoses in adolescents [10–12]. Positive outcomes are found to be the strongest at the level of selective and indicated prevention when based on cognitive behavioral therapy (CBT) or interpersonal therapy (IPT) [10–12]. Next to the results of single level interventions, it is suggested that a multi-modal approach—combining different levels of prevention, for example, public awareness campaigns, gatekeeper education, screening programs, and (preventive) interventions—might be the most effective in reducing depressive symptoms, suicides, and suicide attempts [13–16].

To be able to offer adolescents a preventive depression intervention, adolescents at risk need to be identified and referred if necessary. Screening with standard assessment of depressive symptoms is one way to detect adolescents at risk. It is particularly appropriate when it comes to internalizing symptoms, such as depressive symptoms, that are not verbalized by adolescents and therefore also not easily noticed by parents, schools or healthcare professionals [17–19]. So far, research has mostly focused on depression screening in primary care facilities [20–22]; however schools do also serve as an ideal setting for reaching adolescents who could otherwise be missed by the healthcare system [22, 23]. Despite the promising effects of (multi-modal) preventive interventions, including a combination of screening and indicated prevention [10–16], only a few interventions have been implemented in the existing care services [12, 24]. Therefore, it seems important to continue evaluating and integrating them in healthcare systems.

However, an important bottleneck arises during the implementation of preventive interventions. As in depression treatment [25], participation rates in preventive interventions are often low [26–29]. Previous research in the Netherlands showed that only 1% of the patients with elevated depressive symptoms in primary care make use of the available preventive services [26]. This low participation rate is also experienced within Strong Teens and Resilient Minds (STORM), which is a multi-modal school-based depression and suicide prevention approach that includes screening with a questionnaire, a personal interview with a public health professional, and an effective indicated CBT-based preventive intervention for adolescents with elevated depressive symptoms [27, 30]. For example, based on a systematic screening of 5,222 adolescents in a previous study, 469 adolescents were found eligible for participation in the intervention of the STORM approach of which only 130, or about 27%, participated [27]. This raises the question why only a limited number of adolescents participate in depression prevention programs, despite the fact that the number of adolescents with depression seems to be increasing. To ensure that more adolescents can benefit from prevention, the gap between early identification (systematic screening) and indicated preventive intervention needs to be closed.

One way to close this gap might be by identifying the barriers and facilitators in the process of screening and depression prevention referral in order to improve this process accordingly. Therefore, more insight is needed in this process and in the existing best practices in depression screening. Several factors are found to influence the process of screening and prevention referral. First, there are factors which influence adolescents’ motivation for participation, such as their recognition of symptoms in daily life, beliefs about the outcomes of the intervention, social norms and family beliefs, stigma (e.g., “what might others think?”), practical implications and the preference for individual interventions over group interventions [27, 29, 31–33]. In addition, research shows that parents’ social norms, recognition of symptoms, and experience with depression treatment have an influence on the participation of adolescents [34–36]. However, literature also reveals that when screening results are adequately discussed with adolescents and parents using motivational techniques, adolescents are more likely to participate in recommended services [22]. Besides primary care research indicates that a standardized screening instrument, professional training and a clear protocol, including a protocol about consulting mental healthcare professionals can be beneficial for the management of depressive and suicidal symptoms [20, 37, 38]. These results emphasize the importance of a well- run screening process and the crucial role of professionals who perform screening, particularly their resources and skills. Currently, little is known about how to best execute a screening process in schools, evaluate screening results, make clinical decisions, and, in particular, how to apply motivational techniques with adolescents and parents.

With this study we contribute to existing research by investigating the barriers and facilitating factors in screening for depressive and suicidal symptoms and depression prevention referral within a school-based prevention approach. Considering that professionals have a very important role in the screening and referral process, we will focus on the barriers and facilitating factors they perceive, how they deal with them, and the areas of improvement they consider essential.

## Methods

### Design

This study is a qualitative study. To achieve the research objective, semi-structured interviews were conducted with public health professionals (i.e., nurses and physicians) who execute depression and suicidality screening and prevention referral within the STORM approach.

### STORM approach

Strong Teens and Resilient Minds is a school-based depression and suicide prevention approach, implemented in a rural area in the south eastern region of The Netherlands [27, 30]. Within the approach, there is an extensive collaboration between secondary schools, the Dutch Public Health Service (in Dutch: GGD), and a mental healthcare organization. STORM consists of multi-modal interventions, including systematic screening of adolescents vulnerable for depression and suicidality and an indicated CBT-based group intervention for adolescents with elevated depressive symptoms, located in the school. The process of systematic screening for depressive and suicidal symptoms is part of the routine health assessment of the public health organization among secondary school students from second (13–14-year-old students) and fourth (15–16-year-old students) grades. Students complete a self-reported electronic health questionnaire in the classroom under the supervision of a nurse from the public health organization. When students appear to be at high risk for suicidality the nurse or a physician of the public health organization sees them within 48 h for an emergency personal interview. When students appear to have elevated depressive symptoms, they are invited for a “regular” personal interview with the nurse or physician. Parents are notified by telephone after the interview. If necessary, a referral to follow-up care (e.g., mental healthcare, youth care, general practitioner, or assistant practitioner mental healthcare) is made. Students with elevated depressive symptoms are offered to participate in a preventive intervention, which is a CBT-based group intervention [27, 39].

### Participants and recruitment

All participants were employed by the Dutch Public Health Service and associated with routine health assessment within the STORM approach. Participants were recruited via an email of the management of the Dutch Public Health Service. All participants received a gift voucher of €25 for participation.

A total of 13 (10 nurses, 1 assistant nurse, and 2 physicians; all female), of the approximately 35 public health professionals who are associated with the STORM approach in the region of research, participated. All the participating nurses had completed their nursing education (in Dutch: Verpleegkunde) supplemented with nursing education focused on children and adolescents (in Dutch: Jeugdverpleegkunde); the physicians had studied medicine (in Dutch: Geneeskunde) followed by a course to become a physician in society and health (in Dutch: Jeugdarts); and the assistant nurse had followed education in the field of social work. The participants had different roles in the screening process. The assistant nurse was involved in the practical aspects of screening, and did not conduct personal interviews. The nurses, and in certain circumstances, the physicians, conducted personal interviews (regular and emergency interviews) with the at-risk adolescents, making referrals to follow-up care and offering participation in the preventive intervention. The main task of the physicians in the process was to be available to the nurses for consultation and as back-up. Participant characteristics are summarized in Table 1.

**Table 1:** Characteristics of participants

Characteristics	n(%)
Sex	
Female	13(100)
Professional role	
Nurse Physician Team-assistant	10(76.9) 2(15.4) 1(7.7)
Years of professional experience	
< 5 years 10–20 years 20–30 years 30–40 years > 40 years	1(7.7) 6(46.2) 2(15.4) 1(7.7) 3(23.1)
Years of experience with screening*	
1 year 2 years 4 years 5 years 7 years (i.e., the start of STORM)	2(15.4) 2(15.4) 1(7.7) 1(7.7) 7(53.8)
Training in Dutch guidelines for suicide prevention	
Training followed	11(84.6)
Time spend on screening* each year	
Approximately 3 months a year (1 school) Year round (multiple schools)	11(84.6) 2(15.4)

*screening for depressive and suicidal symptoms

### Interview procedure and qualitative analysis

**Fig. 1:**
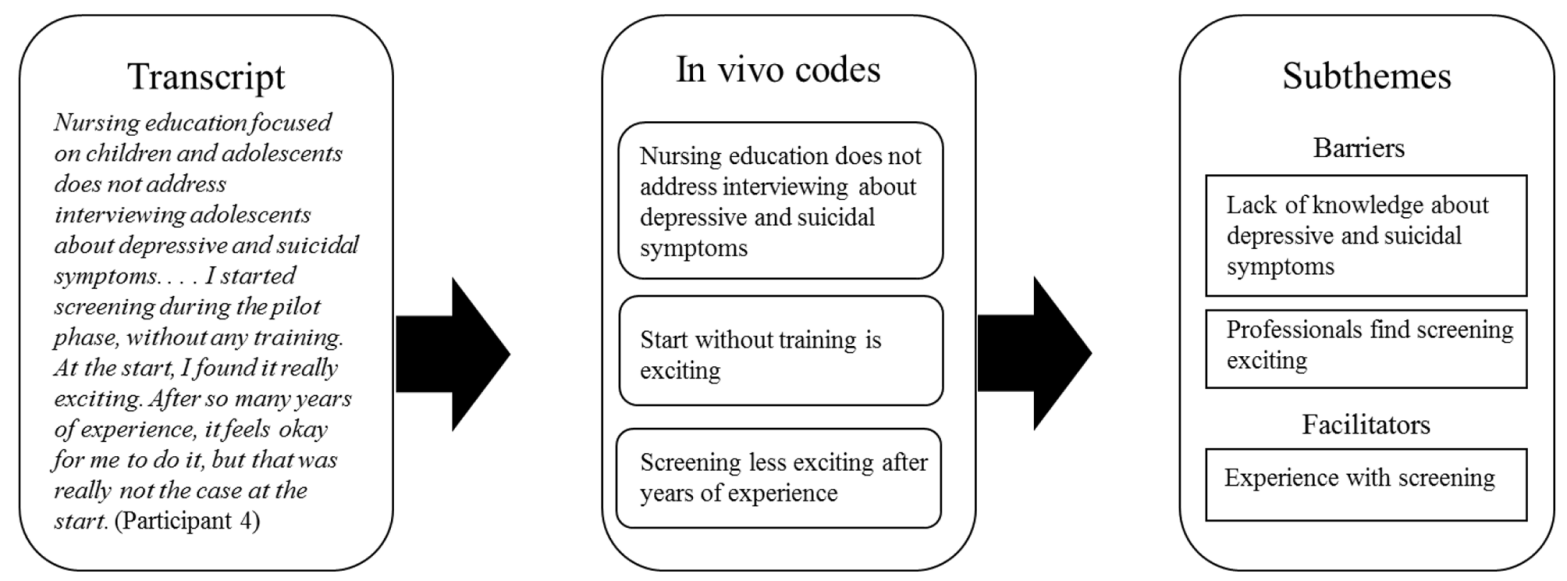
Example of the analysis using the steps of grounded theory *During the open coding phase in vivo codes were extracted from the transcripts. During the axial coding phase, these codes were divided into subthemes. Then, the subthemes were brought together during the selected coding phase to form the main themes (this last step is not shown here).*

The semi-structured interviews were aided by an interview guide (see Table 2).The first author conducted all the interviews. They were conducted face to face, except for two, which were done through video calls. The interviews were recorded and transcribed verbatim for analysis. In line with the steps of grounded theory [40], the study was focused on thematic analysis of the interviews. The verbatim transcripts were coded with the software ATLAS.ti Web (version 22.1.3.0). An example of the coding process is shown in Fig. 1. First, open coding was used to code all relevant fragments of the verbatim transcripts. The coding was done “in vivo” using participants’ own words. Second, axial coding was used to divide the codes into different subthemes. Third, selective coding was used to bring similar and related codes together and obtain a more interpretable structure. Codes were arranged into three main themes, “professional capabilities”, “organization and collaboration” and “beliefs about depressive and suicidal symptoms and participation in prevention”. Each of the main themes consists of subthemes of barriers (e.g., lack of knowledge about depressive and suicidal symptoms), facilitators (e.g., several years of experience with screening) and recommendations (e.g., more extensive training).

**Table 2:** Interview Guide

**Questions**
Participant characteristics: • What is your profession? • What is your educational background? • How many years have you been working in this profession? • How many years have you been screening for depressive and suicidal symptoms? • Was your educational background sufficient for conducting the screening and prevention referral process? Experience in general: • What is your experience with the process of screening and prevention referral? Experience with personal interview and communication with parents: • What is your experience with discussing depressive and suicidal symptoms with adolescents during a personal interview? • What is going well? • What do you find challenging? • What would you recommend (e.g., knowledge, skills or organizational conditions) to meet the challenges? • What is your experience with communication with parents after the personal interview? • What is going well? • What do you find challenging? • What would you recommend (e.g., knowledge, skills or organizational conditions) to meet the challenges? • What is your experience with depression prevention referral? • What is going well? • What do you find challenging? • What would you recommend (e.g., knowledge, skills or organizational conditions) to meet the challenges? • How do you think participation in preventive intervention can be increased?

### Ethics

Ethical approval was granted by the Behavioural Science Institute of the Radboud University Nijmegen, The Netherlands, with reference number ECSW-LT-2022-1-20-7567. All participants participated voluntarily and were informed with an information sheet about the study after they had indicated their interest. Informed consent was obtained prior to the interviews. Data were anonymized after verbatim transcription and both transcripts and recordings were securely stored.

### Validity

Validity and rigor were maximized in several ways. The interview guide incorporated feedback from a content expert (employee within the STORM approach) and an expert in qualitative research. To ensure accuracy in coding, the first and third authors coded the transcripts collaboratively. For the first half of the data, the first author performed the initial coding steps and the third author assessed the coding by examining the applied codes, looking for missing codes, and discussing the disagreements until consensus was reached. For the second half, the roles were reversed. Findings were regularly discussed within the research team. The authors collaboratively developed and refined the three main themes. The findings were presented to three participants and a member of the local management of the Dutch Public Health Service, as a form of “member-checking” [41], to verify the accuracy of the results. The analysis revealed that saturation was reached by the last few interviews.

## Results

Analysis of the interviews revealed three main themes related to barriers and facilitating factors for screening and prevention referral, “professional capabilities,” “organization and collaboration,” and “beliefs about depressive and suicidal symptoms and participation in prevention”. These themes are elaborated below, together with associated subthemes (see also Table 3).

**Table 3:** Barriers and facilitators in screening and prevention referral from the perspective of public health professionals

Barriers	Theme	Facilitators	Recommendations
• Lack of knowledge about and depressive and suicidal symptoms • Lack of experience with adolescents with depressive and suicidal symptoms • Professionals find screening exciting (especially when they first start) • Insufficient opportunities for inter-professional communication and consultation • Management lacks insight in screening activities • Lack of knowledge about the content of the preventive intervention	*Professional capabilities*	• Training in Dutch guidelines for suicide prevention • Additional self-initiated training • Several years of experience with screening for depressive and suicidal symptoms • A supporting network to fall back on	• More extensive training about depressive and suicidal symptoms and personal interviews about these symptoms • A proper introduction program (including job shadowing) • Sufficient opportunities for inter-professional communication, intervision/supervision and consultation • Improving professionals knowledge about the preventive intervention they refer to
• School staff is not informed • Practicalities around screening are not arranged • No permanent trainer at school for the preventive intervention • Practicalities surrounding the preventive intervention are not arranged • Insufficient awareness about the work of cooperating organizations	*Organization and collaboration*	• Practicalities around screening and the preventive intervention are arranged at school • Schools and school personnel take screening seriously • Schools support the preventive intervention and are committed to having as many adolescents with elevated depressive symptoms participate as possible	• Strong collaboration and joint approach between schools (including all school staff) and the public health organization • Contact between trainer and adolescent prior to the start of the preventive intervention. • Commencement of the preventive intervention soon after screening • General awareness of the screening process among cooperating organizations • Close ties between public healthcare and schools and cooperating organizations
• Stigmatizing thoughts of public health professionals • Stigmatizing thoughts of school staff • Negative beliefs (including stigma and taboo) of adolescents and parents: • Denying or downplaying screening results • Not recognizing symptoms • Not open for help • Not wanting a group and/or school intervention	*Beliefs about depressive and suicidal symptoms and participation in preventive intervention*	• Adolescents being open and willing to talk • Parents taking the screening seriously • Parents supporting participation in preventive intervention • Motivational strategies of public health professionals: • Tailored approach during personal interviews • Being directive to ensure cooperation versus respect for autonomy of the adolescent • Linking elements of the intervention to symptoms of the adolescent • Explaining the added value of a group intervention	• More attention to mental health in general and specifically prior to screening (e.g. a mental health lesson for adolescents and parents) • Raising awareness of the preventive intervention (e.g. in a mental health lesson or by referring to it in newsletters or school guides)

### Professional capabilities

The interviews indicated that a majority of the participants felt they lacked the required knowledge and skills to perform the screening process, especially the personal interviews with adolescents. Most participants indicated that performing the screening process on depressive and suicidal symptoms was their first real experience of adolescents with mental health problems. Those who had trained as nurses stated that there had been little or no focus on adolescent mental health problems in nursing education (in Dutch: Verpleegkunde) and nursing education focused on children and adolescents (in Dutch: Jeugdverpleegkunde), which the majority of them had completed. Up to that point in their career, most of the participants had focused mainly on physical health issues in children aged between 0 and 12 years.

*Nursing education focused on children and adolescents does not address interviewing adolescents about depressive and suicidal symptoms. . I started screening during the pilot phase, without any training. At the start, I found it really exciting. After so many years of experience, it feels okay for me to do it, but that was really not the case at the start.* (Chantal)

The training in applying Dutch guidelines for suicide prevention, delivered to them by the mental healthcare organization, was perceived as helpful. However, one day of training was considered insufficient to ensure the required level of professional competence. Several participants also referred to their fear that an adolescent they had seen for a personal interview would attempt suicide or would die by suicide. This distress tends to decrease after a couple of years, but screening continues to be a challenging task for participants.

*When I had just started, I kept walking around wondering whether I had done the right thing. At that time, if I heard that someone had jumped in front of a train, I was really worried that it might be the student I had seen during screening.* (Esther)

In addition, participants felt that high workloads and staff shortages meant there were insufficient opportunities for inter-professional communication and consultation with a physician. Even when such an opportunities arose, they felt the outcome was not always satisfactory, as nurses perceived that physicians in the participating public healthcare organization often lack knowledge and expertise in the area of depressive and suicidal symptoms.

*The physician has a caseload of children between 0 and 19 years old and the workload surrounding 0 to 12 year olds is high, which makes that there is little attention for the 12 plus group. If I need support, which only happens occasionally, they are not sufficiently informed and they return the question to me.* (Joyce)

Participants also claimed that the management in the public health organization was not always adequately aware of screening-related activities and therefore provided insufficient support. The management did not, for example, know how much time a personal interview and a subsequent referral can take, that the professionals partly depended on schools in the performance of their work, and that in this type of work it is necessary to take a break now and then and/or have the opportunity to consult with a colleague. For the reasons outlined above, many participants experienced screening as a stressful process, and some also reported feelings of self-doubt and loneliness.

*I do sometimes feel lonely when performing the screening process. The physician cannot help me because she does not know what to do either, and my close colleague is busy with a case of a student with suicidality of her own.* (Linda)

Participants who indicated that they felt confident about screening typically had several years of working experience with screening for depressive and suicidal symptoms when the interview took place, (self-initiated) additional training, and a professional network to fall back on.

*I do think that now that I’ve been doing it for a couple of years that it is getting easier. Because of the training sessions where we are occasionally informed on “that is how you should do it”, I actually manage to do the interview and ask about complaints.* (Ingrid)

Suggestions for improvement included more extensive training (including a repetition of the practical training in Dutch guidelines for suicide prevention), a proper introduction program (including job shadowing), and sufficient opportunities for inter-professional communication, intervision and/or supervision and consultation.

*You just have to be able to discuss cases with a colleague, like “What do you think?” or “What would you do in this case?”* (Marianne).

In addition to insufficient knowledge about depressive and suicidal symptoms, participants also mentioned insufficient knowledge about the indicated preventive intervention (CBT-based group therapy) as a barrier to effective screening and prevention referral. Several participants indicated that their lack of knowledge about the content of the intervention meant they were unable to explain the benefits of the intervention to adolescents and their parents, making it difficult to motivate adolescents to participate. The participants made several suggestions about how to improve a professional’s knowledge in this regard, for example, by taking a course about the intervention or becoming trainers themselves. Another suggestion was to invite trainers of the intervention to explain the intervention and motivate adolescents for participation.

*If I can only indicate that there is a group intervention that the student can join but cannot explain what this intervention is about, then nobody will participate. It is therefore important to be aware of what adolescents can expect of the intervention. More attention should be paid to this.* (Rebecca)

In summary, the interviews revealed that professionals do not always feel sufficiently equipped in terms of knowledge, skills, and supporting networks. Consequently, they do not always feel well able to execute the processes of screening for depressive and suicidal symptoms and depression prevention referral. More extensive training, a proper introduction program and sufficient opportunities for inter-professional communication, intervision and/or supervision and consultation are recommended by the participants as ways of improving professional competence and self-confidence.

### Collaboration and organization

Participants indicated that the cooperation with the schools where screening takes place is not always optimal. In particular, school staff members are not always properly informed about instances of screening or what the process entails. This can lead to practical problems, such as the lack of a suitable room for the personal interview, as well as incomprehension and resistance among school staff, for example, when teachers perceive personal interviews as a disruption of their lessons.

*One difficulty is that the teaching staff may not know that adolescents experience depressive and suicidal symptoms. If they see a student crying after an interview, they may perceive this as a disruption of their lessons, and they begin to question our working method.* (Linda)

In addition, participants noted that some schools do not fully facilitate the preventive intervention (CBT-based group therapy) in terms of resources and staff. As a result, a permanent trainer may not be available for the intervention at a given school, and, it may be unclear during screening who the trainer will be or when the intervention will commence.

*Until this year, there was no one from the school to provide the preventive intervention, which made it very difficult to get students motivated. There was no driving force from the school. . It is essential to have someone from the school who can represent the training and provide information during screening*. (Ingrid)

This uncertainty about when a preventive intervention will take place (and with whom) was seen as an obstacle to motivating adolescents for participation. The same can be said of long waiting times between screening and commencement of the preventive intervention.

*During screening, I find it difficult that practical aspects of the preventive intervention have not yet been arranged. Adolescents want to know when the intervention will start, at what time, with how many, and who the trainer will be. I cannot answer these questions, and adolescents find this vague.* (Nicole)

Participants experienced the collaboration with a school constructive if the school in question took account of the practicalities, took the screening seriously, and fully supported the preventive intervention.

*I think it is very important that a school supports the screening process and takes it seriously. If necessary, we remove a student quickly from the classroom, and it is helpful if the school supports this. In addition, it is helpful when a school sees the benefit of the preventive intervention and wants to ensure that the intervention group is completely filled.* (Ellen)

To improve the screening process, participants recommended strong collaboration and a joint approach involving both the school (including all the staff) and the public health organization.

*I think it is very important to inform schools properly about the STORM approach. . Many teachers don’t know about the screening process and why it is conducted and also do not know about the preventive intervention. . As far as I know it is communicated on the management level and possibly with care coordinators, but it should spread like an oil slick within the school.* (Linda)

In relation to the preventive intervention, participants advocated contact between the intervention trainer and the adolescent during screening. This would enable the trainer to provide more information about the intervention and allow the adolescent to familiarize themselves with the trainer between screening and commencement of the intervention, making it easier to keep the adolescent motivated. In addition, it was recommended that the intervention should commence as soon as possible after screening to avert the possibility of an adolescent dropping out even before the commencement of the intervention.

Regarding collaboration and communication with other actors, such as mental healthcare organizations, youth care organizations and general practitioners, participants indicated that insufficient awareness of the work done by others can often hinder communication and may delay referral to follow-up care. Suggested points of improvement for a properly functioning screening and referral process included better general awareness of the screening process and closer ties between public health services and other organizations.

*You sometimes notice that GPs are not yet aware of the screening of STORM. I think that is a shortcoming in this time and in this region. The dissemination of the existence of the screening is important. Sometimes you have to repeat a message.* (Esther)

In summary, a lack of knowledge and support in schools was seen to hinder the process of screening and prevention referral. Solutions recommended by the participants include strong collaboration and a joint approach with schools (including the intervention trainer), and also closer cooperation with other organizations.

### Beliefs about depressive and suicidal symptoms and participation in prevention

It emerged from the interviews that various beliefs can influence the screening process. Stigma and taboo were referred to several times in this context. Several participants mentioned that they themselves had stigmatizing thoughts about mental health problems before they started screening for depressive and suicidal symptoms. Some participants indicated that being aware of stigmatizing thoughts that are prevalent in society, and therefore among adolescents and parents, made prevention referral more difficult.

. *. . but I feel that more within myself, in the sense that I think, “It would be helpful for you to participate in the intervention, but I can imagine you do not want all your classmates to know you are not feeling well while everyone else seems well”. I can imagine that adolescents find that difficult.* (Nicole)

Participants also noted stigmatizing thoughts among school staff. For example, teachers who are unaware that depressive and suicidal symptoms can affect adolescents may experience the screening process as disrupting their lessons. It was also indicated that stigma and taboo and other negative beliefs sometimes emerge in conversations with adolescents and parents, as, for example, when they deny or downplay the results of the screening questionnaire.

*There is a type of parent who reacts by saying ‘I already know where this comes from’ or ‘She had this for a long time’ in a certain tone of voice, as if they want to downplay the screening results. I don’t know how to respond to that.* (Laura)

In addition, participants perceived that adolescents are not always open to being helped. They do not always understand the severity of their symptoms, or the possible negative consequences, or they may be afraid or reluctant to seek help.

*There are adolescents who resist follow-up care because they believe they will be able to solve their own problems or because they are afraid to take the next step to follow-up care.* (Chantal)

A lack of motivation to participate in the preventive intervention was also seen to relate to stigma and taboo. Participants noted that adolescents who do not want to participate in a group intervention and/or school intervention seem afraid of what others might think about them and that their problems may become the subject of gossip.

*They think, if I join, I have to talk in a group at school about my feelings. I would rather do this in a setting where others cannot see me.* (Laura)

Finally, participants indicated that because the preventive intervention is not yet as well-known as other school training courses (such as courses that address performance anxiety), this may contribute to the low participation rate and the persistent stigma and taboo surrounding preventive intervention for depressive symptoms.

Although negative beliefs are recurring factors in personal interviews with adolescents, participants also reported that adolescents are often willing to talk and seem relieved after the interview.

*Adolescents are fine with talking about the results of the questionnaires about depressive and suicidal symptoms. They are open about it.* (Joyce)

In addition, many parents take the screening process seriously and like to know about the screening questionnaire results and the subsequent interview. Participants also noted that if parents support participation in the preventive intervention, adolescents are more likely to participate.

*Parents often appreciate it that you call them. They feel that things are going on and/or they are already aware of the complaints of the adolescent. They are grateful that someone is willing to help.* (Rebecca)

To overcome negative beliefs and to increase awareness about the preventive intervention, some of the participating professionals recommended more attention to mental health in general and specifically prior to screening, for example, by a mental health lesson for adolescents and parents, including information about the preventive intervention. Another suggestion was to raise awareness and enhance the normalization of the preventive intervention by referring to it in school guides and/or newsletters.

*Adolescents learn about maths and Dutch language, but it would be good to also have lessons about mental health, I think. . During these lessons one can also show a video about what the preventive intervention entails. No one will think that’s strange. When the screening process then starts afterwards, the picture is complete.* (Tiny)

The interviews also revealed several strategies that public health professionals used to motivate adolescents and parents to participate in preventive intervention. In general, professionals indicated that each conversation is different and therefore requires a tailored approach. They felt it was important to take the time to build trust rather than asking immediately about depressive or suicidal symptoms. To connect with adolescents and parents, they also use words like “not feeling well” or “feeling down” rather than talking about “depression” or “depressive symptoms” and discussed examples of issues adolescents suffer from to make it understandable and personal.

*Some adolescents ask “What is gloomy?” They really don’t know. Then I ask them how they feel. . Or I ask how resilient they are in certain situations. . And I ask specific questions such as “Do you ever think about death?” or “Do you ever hate yourself?”* (Jeanette).

When parents and/or adolescents downplayed symptoms, participants said they would sometimes set this aside for a while before calling a few days later. Sometimes a more directive approach was needed, for example, when it became necessary to involve parents or make a referral to follow-up care or prevention. Participants felt that a directive approach made it easier to secure adolescents’ cooperation and that actions could be taken more quickly than by only asking them to follow professional advice.

*Sometimes it just depends on how you ask the question. If you say, “Do you mind if I inform your parents?” Then they might say “No, rather not.” While when I say, “I always inform parents,” Then they often agree.* (Ingrid)

When making arrangements—for example, about how parents are informed—participants preferred to seek the adolescent’s cooperation as a way of acknowledging their desire for autonomy.

*“Are you going to tell yourself first?” Then I’ll call your parents tomorrow or “would you prefer to do it together?” or “would you like me to call your parents?” I give them the choice to respect their own autonomy.* (Linda)

To motivate adolescents to participate in prevention, it was considered useful to link elements of the intervention to the adolescent’s issues.

*I say something like this to the adolescent: “You say that you deal with things in a certain way, and that you would like it to be different. This training can help you with that by practising and discussing things of this kind with each other. I think this can help you a lot.”* (Esther).

In some cases, it was considered helpful to explain the added value of a group intervention, where you can learn from and support others with the same complaints. Participants also suggested the use of success stories.

*Not everyone likes the group intervention approach to prevention. However, you can explain why it may add value, as participants learn from each other and see that they are not the only ones with mental health issues.* (Chantal)

In summary, the beliefs of public health professionals, school staff, adolescents, and parents—especially, stigma and taboo—can make the process of screening and prevention referral more difficult. To enhance the process, more attention should be paid to mental health and preventive intervention. In addition, a number of communication strategies/motivational techniques can be used to motivate adolescents and their parents to participate in preventive intervention.

## Discussion

The aim of the present study was to examine the barriers and facilitating factors in the process of screening for depressive and suicidal symptoms and depression prevention referral in a school-based depression and suicidal prevention approach in order to identify the conditions that need to be met and the factors that can be intervened on to close the gap between early detection and participation in depression prevention. Although the prevalence of depression is increasing, only a limited number of adolescents participates in prevention. To elucidate this gap, we focused on the perspective of public health professionals in a school-based setting. Three main themes emerged from the interviews, “professional capabilities,” “organization and collaboration,” and “beliefs about depressive and suicidal symptoms and participation in prevention”. Previous research has already shown that beliefs of adolescents and parents and organizational aspects (such as the accessibility of the intervention and whether or not it is a group intervention) influence whether or not adolescents want to participate in prevention [20, 27, 29, 31, 32]. This research using a school-based prevention approach shows that the capabilities of the professionals and the cooperation between different organizations also play a role in the process of screening and prevention referral.

The study revealed that public health professionals do not always feel sufficiently equipped in terms of knowledge, skills, and supporting networks to perform screening on depressive and suicidal symptoms and depression prevention referral. This may indicate that screening depressive and suicidal symptoms requires a different approach than just the monitoring of physical health by public health professionals and public health organizations that they have basically focused on in The Netherlands in the past. The feeling of being inadequately equipped in terms of training appeared earlier in studies exploring adolescent mental health management in primary care [20], paediatrics [42], and even mental healthcare [43, 44]. Despite a decent education in healthcare, the professionals in these settings, just like the public health professionals in this school-based prevention setting, do not always feel confident in guiding adolescents with depressive and suicidal symptoms. Depressive and suicidal symptoms seem to differ from other mental or physical complaints in this account [42, 45]. In particular, signaling and treating suicidal symptoms evokes distressing reactions such as anxiety, panic and doubts about professional competence because of the negative impact a suicide can have on the reputation of a professional and a mental health organization [43, 44]. In line with mental health professionals, who report consulting a supervisor or informally supporting each other as strategies to cope with their distress and to share responsibility [43, 44], the public health professionals in this study would also prefer more possibilities for inter-professional communication, consultation and support—a holding work environment [46]. It may be that a holding work environment—holding work environments are defined by strong interpersonal or group-based relations that enable self-reliant workers to manage situations that trigger anxiety [46]—can help public health professionals to experience less stress in performing the screening and referral process and feel more comfortable and confident. In addition to sufficient options for training and professional development, it might be important for future prevention approaches to create a holding work environment for professionals who perform the process of screening and prevention referral. Future research should demonstrate whether this would lead to actual improvements.

In addition to insufficient support within the public health organization, a lack of good cooperation with external organizations can hinder screening and prevention referral. The activities of the public health professionals are part of the STORM approach. Within this approach there is a cooperation between public healthcare, schools, and mental healthcare to detect and address depressive and suicidal symptoms at an early stage. In the literature, this is known as collaborative care, a cooperation of professionals to improve the reach of (mental) health services and facilitate positive (mental) health outcomes for individuals by combining different interventions [47, 48]. Despite the solid set up of this approach, this study shows that there is still room for improvement in the cooperation between schools and the public health organization. Schools may have to become used to the existence of mental health as part of their curriculum and accept that they have to make time and space available for the associated activities. [32, 52]It may be that when there is strong collaboration between public health organizations and schools and a joint approach is taken by them, practicalities will be easier to arrange, and it will be easier to motivate adolescents to participate in preventive intervention. As suggested by previous research [49], in order to bring about the full involvement of schools and school staff, it may be important that all staff know about the entire process of screening and preventive intervention and that the public health professional and the trainer of the preventive intervention are familiar faces within the school. In addition, in line with earlier research [50], public health professionals in the current study indicated that insufficient awareness of the work of other organizations—for example insufficient awareness of the screening process by mental health professionals—, can hinder communication and may delay referral to follow-up care. Therefore, it seems also important that co-operating organizations in prevention approaches are aware of each other’s activities and maintain close ties.

The participating public healthcare professionals recognized negative beliefs in themselves and among the school staff, adolescents and parents. As negative beliefs about depressive and suicidal symptoms and preventive intervention seem to be reflected in all actors in the process of screening and prevention referral, more knowledge about mental health may be needed across the entire society. Previous research on mental health literacy, help-seeking behavior, and stigma reduction found promising results from adolescent-targeted awareness and education programs [51, 52]. Little is known about promoting parents’ mental health literacy. However, the professionals in this study do suggest that parental mental health education could be helpful. As in previous research [34–36], they perceive that parents can play an important role in adolescents’ decision about participation in preventive intervention. Both adolescent and parent mental health literacy programs are currently piloted within the STORM approach. Gatekeeper training [53] and training in Dutch guidelines for suicide prevention [54, 55], may contribute to reducing stigmatizing thoughts and increasing knowledge about preventive intervention in school personnel and public health professionals, respectively. Incidentally, both are part of the STORM approach [30].

In addition to education strategies to increase mental health literacy and increase help-seeking behavior, the current study reveals a number of communication strategies for professionals that are seen as helpful in motivating adolescents for participation in preventive intervention. In line with previous studies [49, 50], public health professionals indicate that a tailored, person-centered approach is required for each personal interview and conversation. To connect with adolescents and parents, it is helpful to adapt language and give many examples of the issues an adolescent suffers from, next to taking the time and building trust. Contrary to adolescents’ preference for self-reliance [50], public health professionals prefer a directive approach to secure adolescents’ cooperation. While adolescents have a desire for autonomy, professionals may have an incentive to take actions, such as involving parents or referring to follow-up, as quickly as possible to be able to share responsibility with others. Public health professionals recommend seeking the cooperation of adolescents when making arrangements—for example, about the way parents will be informed—with which professionals seem to create a balance between being directive and respecting autonomy. A helpful strategy to motivate adolescents for participation is linking symptoms of the adolescent to elements of the preventive intervention. [53]Another strategy is explicitly explaining the benefits of a group intervention, which is in line with the positive experiences that adolescents report after participating in group interventions, like experiencing that they are not alone in their complaints and that they are given the opportunity to learn from and support each other [56]. Before integrating these strategies into training programs for professionals, future research should determine whether these strategies do lead to actual improvements in motivating adolescents for participation in prevention.

### Strengths and limitations

An important strength of our study is that we used a different approach to study the process of screening and prevention referral from the more usual quantitative studies on the reach of prevention. The interviews gave us the opportunity to thoroughly investigate this process and the barriers and facilitating factors. Further, in contrast with many previous studies that focused on adolescent and parental factors and primary care setting, we specifically focused on factors related the public health professionals in a school-based setting. This provides a more complete picture of the process of screening and prevention referral in a school-based prevention approach and makes it possible to give concrete advice and make recommendations based on which adjustments can be made in practice in the future.

In addition, there was a broad range of work experience within the group of participants, which is a good reflection of the team composition of public health professionals within STORM. It turned out that more experienced professionals often still encounter the same difficulties as less experienced professionals. Another strength is that this study was conducted within an implemented depression and suicide prevention approach and could reveal the barriers and facilitating factors that have not yet emerged in a research setting.

This study also has some limitations. Despite the fact that the participants in this study work in different teams, this study took place within one public health organization and a specific prevention approach (the STORM approach) in a rural area of the Netherlands, which may limit the generalizability of the findings. In addition, all participants were female and although the profession of public health professional is mostly a female profession in the Netherlands and there are only women employed within STORM at the moment, this may make results less generalizable to other international settings. Follow-up research should examine whether the results can be generalized to other settings, team compositions, areas and countries. Finally, this study was explorative in nature. Future research is needed to find out whether the recommendations of this study actually lead to the expected improvements.

## Conclusion

This study found three main themes influencing screening for depressive and suicidal symptoms and depression prevention referral in a school-based depression prevention approach, focusing on the perspective of the professional: “professional capabilities,” “organization and collaboration,” and “beliefs about depressive and suicidal symptoms and participation in prevention.” To further enhance the process of screening and prevention referral in a school-based setting performed by public health professionals, this study suggests sufficient opportunities for enhancing professional competence and, even more important, a holding work environment for professionals, a strong collaboration and a joint approach with schools and close ties with other cooperating organizations, broad education about depressive and suicidal symptoms and preventive intervention, and education in specific motivational techniques for professionals. Future research should point out whether these suggestions actually lead to closing the gap between early identification and successful preventive intervention.

## Data Availability

The data generated and analyzed during the current study are not publicly available as they contain information that could compromise participant privacy but anonymized data could be made available for the purpose of research by the corresponding author on request, based on the merit of the case. The STORM approach is funded by (local) government in the Netherlands. Schools do not have to pay for participation in the prevention approach. The participating professionals might have had an interest in the study because of the potential improvements that could result from the study. The authors declare that they have no competing interests.

## Abbreviations

CBT: Cognitive behavioral therapy
STORM: Strong Teens and Resilient Minds
